# Does the COVID-19 war metaphor influence reasoning?

**DOI:** 10.1371/journal.pone.0250651

**Published:** 2021-04-28

**Authors:** Francesca Panzeri, Simona Di Paola, Filippo Domaneschi

**Affiliations:** 1 Department of Psychology, University of Milan–Bicocca, Milan, Italy; 2 DAFIST–Laboratory of Language and Cognition, University of Genoa, Genoa, Italy; Sant’Anna School of Advanced Studies: Scuola Superiore Sant’Anna, ITALY

## Abstract

In recent times, many alarm bells have begun to sound: the metaphorical presentation of the COVID-19 emergency as a war might be dangerous, because it could affect the way people conceptualize the pandemic and react to it, leading citizens to endorse authoritarianism and limitations to civil liberties. The idea that conceptual metaphors actually influence reasoning has been corroborated by Thibodeau and Boroditsky, who showed that, when crime is metaphorically presented as a beast, readers become more enforcement-oriented than when crime is metaphorically framed as a virus. Recently, Steen, Reijnierse and Burgers replied that this metaphorical framing effect does not seem to occur and suggested that the question should be rephrased about the conditions under which metaphors do or do not influence reasoning. In this paper, we investigate whether presenting the COVID-19 pandemic as a war affects people’s reasoning about the pandemic. Data collected suggest that the metaphorical framing effect does not occur by default. Rather, socio-political individual variables such as speakers’ political orientation and source of information favor the acceptance of metaphor congruent entailments: right-wing participants and participants relying on independent sources of information are those more conditioned by the COVID-19 war metaphor, thus more inclined to prefer bellicose options.

## Introduction

The severe acute respiratory syndrome coronavirus 2 (SARS-CoV-2) was first identified in December 2019 in Wuhan, China. On February 10^th^, 2020, the Chinese leader Xi Jinping vowed to win the people’s *war* against the novel coronavirus [1]. One month later, in his address to the Nation, President Emmanuel Macron repeated “Nous sommes en *guerre*” (“We are at war”) six times [2], and was soon followed by British Prime Minister Boris Johnson and by US President Donald Trump, who presented themselves as *wartime* leaders [3, 4]. War metaphors were particularly pervasive in Italy, the first Western country to have a major outbreak of COVID-19. The use of bellicose metaphors to describe the coronavirus pandemic invaded political speeches and journalistic discourses: on the 25^th^ of March, Mario Draghi (former president of the European Central Bank) declared “we face a *war* against coronavirus and must *mobilize* accordingly” [5]; on the 16^th^ of March, the then Italian Prime Minister Giuseppe Conte remembered all the Italians who were fighting “in the *trenches* of the hospitals” [6]. The reference to a wartime was echoed in many other occasions by Domenico Arcuri (Special Commissioner for the COVID-19 Emergency) who, on the 22^nd^ of March, declared “We are at *war*, we have to find the *weapons*” [7], and by the leader of the right opposition party, Matteo Salvini, who on the 12^th^ of March, while commenting the “*war* bulletin” of the death count, claimed: “During *wartime*, *war* measures must be adopted” [8].

The use of war metaphors is a widespread strategy in public speech for framing and representing the challenges to be faced. US Presidents declared war against poverty (Johnson, 1964), crime (Johnson, 1965), drugs (Nixon, 1971), cancer (Nixon, 1971), inflation (Ford, 1977); but this metaphor is so ubiquitous that it can also involve apparently not belligerent enemies such as traffic jams, sunshine and even salad [9]. A study examined all articles published on three magazines (TIME, Newsweek and the Canadian Maclean) over a period of twenty years (1981–2000) and found that war/battle metaphors occurred in 15% of the articles [10].

Even though extremely pervasive, these metaphors have often been depicted as misleading, if not harmful, and the same criticism was raised against the COVID-19 bellicose rhetoric: presenting the pandemic as a conflict might be dangerous, because metaphorical references to warfare are claimed to induce negative consequences on the way the audience conceptualize the epidemic situation, and eventually on the way they will react to it. After a scrutiny of these criticisms (§ “The war on war metaphors”), we discuss the theoretical approaches that lie at their heart as well as the experimental studies that support them (§ “Metaphors we are convinced by”). Then, we present a study aimed at verifying whether participants’ preferred solutions to coronavirus-related problems were influenced by the presence of bellicose metaphors (§ “The study”). Afterwards (§ “Discussion”), we present the data collected and explain that the results of the study do not support the idea of a direct effect of the war metaphor on participants’ thoughts and actions. Rather, socio-political individual variables such as speakers’ political orientation and source of information favor the acceptance of metaphor congruent entailments. Finally, based on the results of the study, we conclude that the battle to eradicate war metaphors from public speech might be not only hopeless, given their ubiquity, but also useless.

### The war on war metaphors

Since December 2019, coronavirus and the related disease (COVID-19) quickly spread all around the world, causing more than 100 million infections and over two million deaths (January 2021). The leaders of the first infected countries faced the problem of explaining to the population the threat posed by this disease and the necessary precautions that everyone had to put in place to slow the contagion rate. The virus is, obviously, invisible, and the initial symptoms of COVID-19 are flu-like: the difficulty was then to find a strategy to convince the population of the risks of this unknown virus. Drawing a parallelism between abstract concepts we are unfamiliar with and more tangible events we have experience of permits to acquire knowledge and to understand otherwise inaccessible concepts. The transmission of a virus (among people, and inside a person’s body), and the development of a disease are invisible processes hard to conceptualize: through metaphors, these complex and abstract issues are linked to relatively simpler experiences. In particular, the war metaphor permits to think of viruses as (invisible) enemies that attack our cells and of medicines as weapons that can defeat the invader. Furthermore, the pandemic caused by SARS-CoV-2 seriously alarmed the world population, and the bellicose rhetoric provided “a clear and simple explanation of the threat and how to respond to it” [11: 1]. Since many war related concepts can be used to talk about illness, this constitutes a “structural metaphor” in Lakoff and Johnson’s terms [12]. The source domain “war” provides a rich cluster of notions that might be associated with the target domain “pandemic”: there are several structural correspondences, such as “the virus and an enemy; health professionals and an army; sick or dead people and casualties; eliminating the virus and victory” [13: 51]. In addition, when leaders resort to the war metaphor to talk about COVID-19, they might effectively obtain positive consequences: they “convey a sense of urgency and emergency”, alerting the population on the seriousness of the threat, and thus they succeed in communicating “the need for everyone to mobilize and do their part on the home front”–for citizens, “taking social distancing orders and hand washing recommendations seriously. For businesses, that means shifting resources toward stopping the outbreak, whether in terms of supplies or manpower”. Moreover, politicians can take advantage of the war metaphor “to cut through partisan disagreements and unite people against a common enemy” [14]. Indeed, the war metaphor has been extensively used to frame the pandemic issues, not only by politicians, but also by Twitter users [15].

Even though presenting the pandemic in military terms can have positive outcomes, many newspaper articles all around the world recently raised important concerns about the use of the war metaphor. This was so much so that a group of researchers launched an initiative, #ReframeCovid, aimed at collecting alternative metaphors to talk about COVID-19 (see also [13] for a defense of the fire metaphor). Two different lines of criticisms can be recognized. On the one hand, it is claimed that the link between the pandemic and the war cannot be pushed too far, since they share only superficial similarities. The virus might be conceptualized as an (invisible) enemy, but it surely does not have any “intention” and cannot sign an armistice. Moreover, viewing hospitals as warzones, and doctors as heroes who fight the virus, does not constitute a fair simile, since doctors are employees paid to do their job, but not to risk their lives, and hospitals ought to be safe places where security is always in place.

Besides these concerns related to the plausibility of some correspondences, a more fundamental objection is raised: in a nutshell, the idea is that the linguistic presentation of the pandemic situation as a conflict would carry over not only cognitive consequences, but–more importantly–behavioral implications connected to real wartime contexts. Let us first sketch the argument as it is presented in the media, and eventually ground it in the theoretical and experimental literature (§ “Metaphors we are convinced by”). The media fear that if the pandemic situation is metaphorically presented as a conflict, then the wartime frame will be activated, together with all the related salient entailments. Moreover, not only these entailments will become cognitively relevant, but they will also lead individuals (not only to think, but also) to act accordingly. Thus, for instance, if the virus is presented as an enemy, other conceptually related propositions will activate as well: citizens become “soldiers” in a conflict, politicians “call for obedience rather than awareness and appeal to our patriotism, not to our solidarity”, and this might favor “shifts towards dangerous authoritarian power-grabs” [16]. Since war is inherently divisive, with allies and enemies, patriots and deserters, when the pandemic is described with a bellicose language, the interlocutors will be led to adopt “a good ‘us’ versus bad ‘them’ mentality” [17] and go after whoever is felt as a threat–urban inhabitants (since in many Nations the SARS-CoV-2 first spread in big cities), Asiatic people (because the virus was first identified in China), and so on. Again, presenting hospitals as frontlines “makes a desperate appeal to the necessity of chaos” [18], and this justifies the structural deficiencies in the health system that did not provide enough personal protective equipment (PPE). As a consequence of this, not only doctors-heroes are expected to fight against the virus without enough weapons-PPEs, but more in general the preventable mortalities become fatal casualties, since “as in an actual war, collateral damage [are deemed] to be unfortunate but inevitable” [19].

To sum up, there has been a great deal of criticism against the use of the war metaphor to talk about the pandemic. In the next section, we now review the theoretical accounts and the experimental studies that support these worries.

### Metaphors we are convinced by

The idea that conceptual metaphors, and in particular military metaphors, may have an impact on language users’ behaviors, on their social attitudes and decisional processes has been explored by Susan Sontag [20, 21] in her original works about the language used in medical discourse to describe illnesses and people affected by diseases such as cancer and HIV. Sontag [20] argues that the military rhetoric, for instance about cancer, contributes to stigmatize a disease as well as its bearers. In her view, the figurative representation of “cancer as a war”, in which the body is framed as a battlefield, illness as an unavoidable causality or as an enemy to fight and in which patients are represented as soldiers, contributes to consolidate the general perception of the disease as a punishment and as something fatal and shameful to repress and defeat–on the pervasiveness of the bellicose vocabulary in medical discourse, see also [22]. The undesired consequence of this rhetoric, she argues, is the reinforcement of the idea that cancer patients, as soldiers, bring some responsibility on their shoulders in fighting the cancer/war and, consequently, that certain personality types are by default expected to be more prone to resist and fight cancer than other personality types.

Since Sontag’s seminal work, several studies have tried to provide theoretical and empirical evidence corroborating the idea that, compared to other conceptual metaphors (e.g., a “disease is a journey”), the war metaphor contributes to affect patients’ emotional reactions to a disease (cf. [23]). In this respect, interestingly, Flusberg and colleagues [24] argue that conceptual metaphors, like the war metaphor, help to efficiently structure and communicate new knowledge and to urgently express a negative emotional tone that both captures language users’ attention and motivates their action. Yet, they claim, the war metaphor is essentially dependent on the context of use, such that its adoption can elicit positive or negative outcomes, depending on the circumstance. For instance, while in certain situations the conceptual war metaphor may favor the adoption of positive behaviors of cancer prevention, other uses may have the opposite effect of representing the disease as an enemy to fight in a battle; a representation that can trigger in both patients and non-patients a sense of threat, fear, panic and demotivation. In this respect, Hauser and Schwarz [25, 26] further supported this conclusion by showing that these sorts of violent metaphors can affect negatively non-patients’ perception and judgment of cancer treatment and prevention. When represented as a battle, cancer treatment is more likely to be perceived as something difficult to pursue and as an enterprise doomed to failure: the fear of the fight may discourage some form of prevention and increase pessimistic and fatalistic attitudes. Bellicose metaphors and the occurrence of a military lexicon for cancer (e.g., *fight*, *war*, *enemy*, etc.), they conclude, can influence non-patients’ health beliefs and make them less willing to enact healthy behaviors–see also Gustavsson’s work [27] on the use of the war metaphor as an instrument to threat vulnerable population about the use of drugs. In conclusion, there is wide consensus that the war metaphor on cancer is a rhetorical solution to be stigmatized and abandoned and that health care professionals, patients and more generally medical discourse need new narratives–see [28, 29].

It is important to stress that Sontag’s observations on the risks of using bellicose metaphors to talk about illness have been interpreted in two distinct ways. On the one hand, scholars in the field of rhetoric and literature studies view metaphors as clusters of associations that permit to provide meaning to unfamiliar situations by drawing comparisons to familiar ones. In doing so, they offer a coherent view that highlights some, and inhibits other, thoughts, judgments and actions. Thus, for instance, when cancer is presented as an enemy, the cluster of associations connected to the war frame will make more salient the concept of *fighting a battle* with it, that might end with a *victory* or with a *defeat*; whereas the idea of living with cancer is overshadowed. Importantly, these ideas and ensuing conducts are favored, but not determined, by the presence of a metaphor. Moreover, contextual factors play a predominant role: metaphorical frames might be more or less convincing depending on their intrinsic properties– see for instance [11] that objects to Trump’s war rhetoric its incoherence–and on the audience’ s personal characteristics.

On the other hand, according to Lakoff and Johnson’s Conceptual Metaphor Theory [12], which is developed within the framework of cognitive psychology, structural metaphors determine people’s way of thinking and acting. In this view, linguistic metaphors such as “cancer is an *enemy* in a *war*” guide language users’ thought, their opinion formation and, more importantly, their behavior [30]. For example, Thibodeau and Boroditsky [31] ran five experiments to investigate whether the instantiation of a metaphor impacted on the way people attempt to solve social problems such as crime and gather information to take well-informed decisions. In order to do so, they provided participants with a series of written vignettes that described a crime (only) metaphorically as either a beast or a virus, and asked them to formulate possible solutions to a city crime problem that could vary in terms of degree of enforcement–e.g., enacting socio-economic reforms such as eradicating poverty and improving education vs. catching criminals and endorsing harsher enforcement laws. Data collected suggest that the metaphorical framing contributes to trigger frame-consistent statements that prompt structurally consistent decisional inferences. For example, the two different metaphors influenced participants’ inferences about the crime problem, and contributed to suggest different causal interventions to solve the problem: participants were more inclined to fight back against a crime/beast by increasing the police force, while they were more likely to diagnose and treat a crime/virus through social reform.

In a follow-up work, Thibodeau and Boroditsky [32] further explored the effect of conceptual metaphors on people’s opinion formation and confirmed the previously observed results. In this case, instead of asking participants to produce an original solution to a given crime problem autonomously, they provided a list of alternatives (reform- or enforcement-oriented) within a selection task, and asked participants to select their preferred solutions. They found that, even with those participants that were not explicitly aware of the occurrence of the metaphor in the text, the metaphorical framing covertly affected people’s reasoning by guiding their choice. Both groups chose more metaphor congruent options as compared to the other one: those who read the crime/beast text chose more enforcement-oriented options, while those who read the crime/virus text opted more for reform-oriented options. Hence, data collected support the conclusion that the use of a metaphor can influence not only the first solution that comes to the speakers’ mind but also their decision about the best solution to adopt among a set of given alternatives. Furthermore, interestingly, the metaphorical frame effect seems more powerful when the frame is instantiated early, i.e., when the first metaphor is encountered early in the text [33].

Steen, Reijnierse and Burgers [34] criticized the idea that the use of certain conceptual metaphors may influence the way people reason about an issue, describe it, take decisions about it and act accordingly. The authors offer a critical view of Thibodeau and Boroditsky’ works, showing that their data leave room for alternative explanations that do not directly support the thesis that natural language metaphors inevitably influence people’ s reasoning. Steen and colleagues reported the results of four experiments based on Thibodeau and Boroditsky’s rationale and using an adapted version of their original materials. This included a version of the text without other potentially confounding metaphors and, more importantly, a neutral control condition that did not contain metaphorical terms. Moreover, in two of their experiments (Experiment 1 in Dutch, and Experiment 2 ran in the United States), they also collected pre-exposure measures, asking participants to indicate their preference for (general) crime reducing policies, presenting the five options (three enforcement-oriented and two reform-oriented options). These options were later presented as possible solutions to the specific crime problem framed with the beast metaphor, with the virus metaphor or with no metaphor. Interestingly, *contra* Thibodeau & Boroditsky, with such methodological adjustments, the authors found no metaphorical framing effect. Yet, they observed that the exposure to a criminal content may affect people’s reasoning: participants’ choices of enforcement-oriented solutions increased after reading a text about a crime problem in a city, independently of the problem being presented in metaphorical terms (as a beast or as a virus) or in neutral terms.

In discussing their data, and the differences with Thibodeau and Boroditsky’s results, Steen and colleagues hypothesize the influence of other potential mediators and moderators that might intervene, favoring or blocking the derivation of metaphor congruent inferences, such as participants political opinions, and their being competent on an issue. Indeed, Burgers, Konijn and Steen [35] claim that a key issue concerns precisely the individual-level effects of frames, and they invite researchers to investigate the conditions under which a metaphorical frame impacts the audience’s own stance on that issue, “identifying mediators and moderators under which such individual-level effects are increased or hampered”. Still, it is important to stress that there is not yet consensus at all on what individual factors influence the metaphorical framing effect.

### The study

We have discussed how the frequent appeal to bellicose language to frame issues related to COVID-19 has been widely criticized, under the assumption that the war rhetoric might induce the audience to think and act in metaphor congruent ways. These ideas are grounded on the cognitive psychological approach to metaphors that is typical of the Conceptual Metaphor Theory. This account assumes that reference to structural metaphors leads to the activation of other congruent entailments and that these additional propositions shape the interlocutors’ thoughts and influence their behavior. At the same time, there are reasons to think that individual characteristics might amplify or reduce the metaphorical framing effect. In this respect, it has been claimed that the issue “should be rephrased as a question about under which conditions metaphors do or do not influence our reasoning and, in particular, about “which metaphorical frames influence which types of people under which conditions” [33: 23]. In this work, we pick up on this suggestion. The goal of this study is two-fold. First of all, we aim at investigating whether the COVID-19 war metaphor has a framing effect, that is, if presenting the COVID-19 pandemic as a war–as compared to a non-metaphorical condition–affects people’s reasoning in terms of opinion formation. Moreover, we intend to explore if some groups of people are more sensitive than others to the exposure to a metaphorical framing.

This study was run in Italy in June 2020. Italy recorded the first local case of COVID-19 on the 21^st^ of February 2020; one month later, on the 28^th^ of March, Italy (a country with 60 million inhabitants) surpassed the number of infections in China, with 86 498 cases. Even if a lock-down was decided in the beginning of March, the contagion rate continued to grow and reached more than 230 000 cases and more than 33 thousand deaths by the end of May. When the experiment was distributed (June 4–26), Italy had just re-opened activities and regional borders, after almost two months of strict lock-down.

#### Experimental hypotheses

Our aim was to investigate whether the presentation of the COVID-19 pandemic within a war metaphorical frame influences participants’ disposition to endorse particular options that are claimed to be connected with military situation. To achieve this goal, we presented participants with scenarios that were either neutral (i.e., no metaphorical expressions) or included bellicose metaphorical terms, and we asked them to choose the options they agreed more with. If–as conceptual metaphor theorists claim–the war metaphorical frame influences people’s willingness to form specific beliefs and act accordingly, we expect the participants who saw the war metaphorical frame to be more inclined to favor military-like actions as compared to those who were exposed to the neutral version of the texts. Our second goal was to control for the possible role of individual factors in amplifying or reducing the metaphorical framing effect. Since there is no consensus on the literature on what factors might influence the framing effect [35], our hypotheses in this respect are more exploratory than confirmatory. We decided to investigate (besides classical demographic variables, such as age, gender, educational level) political orientation and the appraisal of political figures, on the one hand, and the preferred source of information, on the other hand. As for partisanship, some studies [31, 32, 36] found that (right-wing oriented) conservatives were less susceptible than (left-wing oriented) liberals to the influence of metaphors, but at the same time contrasting results have been obtained with reference to the level of political knowledge (see [35] for an overview). Moreover, since a war frame conveys a sense of urgency that justifies authoritarian decisions (see, among other, [13, 16]), we hypothesized a possible link between participants’ appreciation of the people in charge during the pandemic in Italy and their endorsement of metaphor congruent options. As for participants’ preferred source of information, we were interested in finding possible links between the type of information channels (i.e., traditional vs. unconventional ones; the former being typically a more serious and complete source of information, and the latter constituting a source of information more emotionally loaded and possibly inaccurate) and the choice of war congruent entailments.

## Methods

### Participants

Two hundred thirty-one participants took part in the experiment (mean age: 33.26; SD: 14.72; age range: 18–66; F: 149). They were all native speakers of Italian; some of them were students in the Department of Psychology of the University of Milan—Bicocca and received credits, whereas the majority of them participated on a voluntary basis after receiving an invitation through social media. Written informed consent was obtained from all participants prior to the beginning of the experiment. Participants were not aware of the goal of the experiment. Since the task was implemented online, some participants did not complete the experiment. Those who did not reach at least the 80% of the test were excluded from statistical analyses. Based on this criterion, 29 participants were excluded from the analyses. The final sample included therefore data from 202 participants (mean age: 33.53; SD: 14.77; age range: 18–66; F: 129). The study was approved by the Research Ethics Committee of the Department of Psychology of the University of Milan—Bicocca.

### Materials and procedure

We created a questionnaire that was composed of three parts: (A) a series of socio-demographic questions; (B) six short texts that presented an issue related to the pandemic situation and after which participants had to choose the options they agreed more with; (C) a series of questions concerning political opinions and the preferred sources of information.

In the first part (A), after reading the Informed consent, participants were asked their gender, age, educational level, and the Region of Italy in which they were living.

The central part of the questionnaire (B) investigated the possible influence of the metaphorical framing on participants’ disposition to prefer particular options for the resolution of a problem. It consisted of six short scenarios connected to the pandemic situation: the criteria for the world distribution of the future vaccine; the opportunity of forcing the use of an App to track the contagion; the issue on whether or not limiting the liberties of particular groups of persons who might spread the virus; the discussion about funds distribution and people’s circulation within the European Union; the situation in the hospitals during the peak of the pandemic, when doctors and nurses had to treat patients without enough PPEs; the spreading of inaccurate, if not blatantly false, news about the correct way to face the contagion. There were two different versions of the six texts: one did not contain any metaphorical expression (Neuter); the other one contained several (4 to 6) metaphorical expressions related to the war frame.

**Fig 1:**
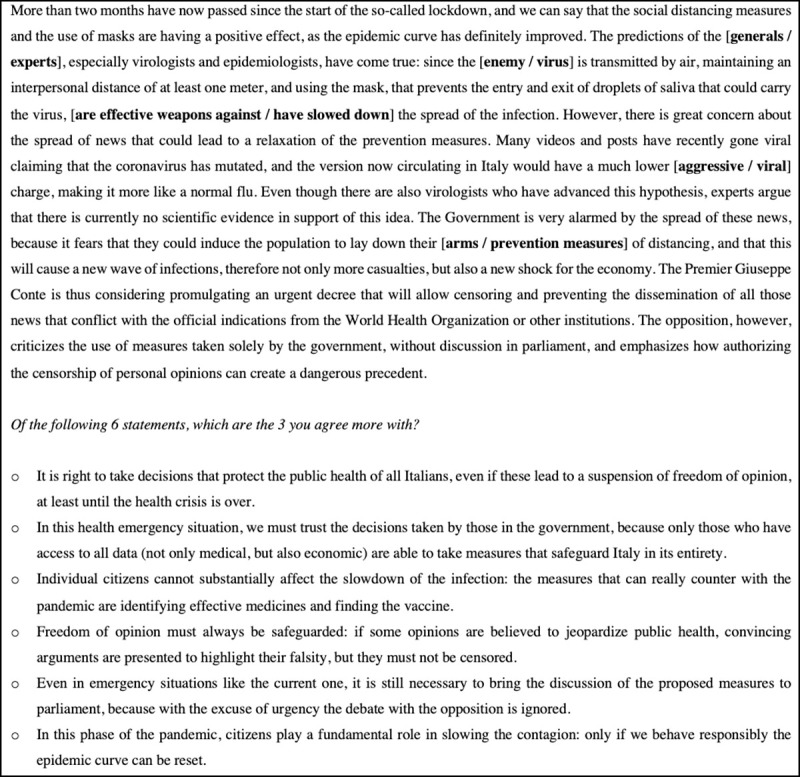
Transliteration in English of the scenario “Fake News” with the relative options. The metaphorical and the neutral text differed in the expressions in bold displayed within square brackets.

After reading the text, participants were presented with six options (presented in random order), and they were asked to choose the three options they agreed more with. Three of these options were congruent with the warfare scenario. For instance, as already mentioned in the introduction, the bellicose language is claimed to justify authoritarian governments, that may have the opportunity to seize more power, be legitimized in infringing personal liberties, and delegate to “generals” the decisions, therefore removing responsibility from the civilian [13, 16, 37–39]. Thus, in our experiment, after a text on the spreading of fake news, the three war congruent options had to do with participants’ willingness to (temporarily) suspend personal liberties, to trust government decisions, and to delegate to “experts” the handling of the pandemic; whereas the other three options made contrary claims (always safeguarding civil liberties, requiring the discussion in parliament of all the measures, individual responsibility for slowing the contagion)—Fig 1.

The six available options, then, were three pairs of contrary claims: in the discussed example, option 1 was in favor, and option 4 was against, the suspension of civil liberties; decisions had to be taken autonomously by the government in option 2, and by discussing with the opposition in option 4; in controlling the contagion, citizens’ behaviour is presented as irrelevant in option 3, and as fundamental in option 6. As a consequence of this, to provide consistent answers, participants could not select the contrary claims of the same pair.

In the final part of the questionnaire (C), participants were first asked to indicate how often (on a 5-points scale, from 1-never to 5-always) they were relying on the following sources to obtain information: news or programs on television; (online or print) traditional newspapers; independent information channels; and social media. Notice that in Italy, as in many other Countries, so-called independent information channels represented the sounding board for unchecked news and conspiracy theories to be spread. Participants were then asked to indicate their political orientation, on a (horizontal) 7-points scale, in which 1 corresponded to “left”, 4 to “center” and 7 to “right”. Finally, they were required to evaluate on a 10-points scale (i.e., 1-very bad, 10-excellent) how some political figures (i.e., the Premier, the national government, the opposition, and their Region governor, who is in charge of the local sanitary system) managed the COVID-19 emergency.

Participants were randomly assigned to one of two experimental conditions of part (B) of the questionnaire, namely the metaphoric (N. 6 trials/passages) and the neutral (N. 6 trials/passages) condition. Part (B) of the questionnaire was therefore a between-subjects design. Of the final sample of 202 participants, 106 participants saw the experimental material in the metaphoric condition and 96 in the neutral condition.

The questionnaire was administered online using Qualtrics_®_^XM^.

### Coding

In the metaphor task, for each passage/trial, we collected each participant’s selection of the 3 statements (out of 6) they agreed more with. Our dependent variable was the number of war congruent options selected by each participant, for each trial/passage in the metaphoric and neutral conditions. The coding procedure underwent several steps and consisted in what follows. First, for each trial, each participant’s choice of a war congruent statement was coded as 1; each participant’s choice of the contrary (and thus “war incongruent”) statement was coded as 0. Second, since the 6 choices actually consisted of 3 pairs of contrary claims (see Materials and procedure), for each trial, we checked whether the responses were consistent: a participant’s response was considered inconsistent if, within the same trial, the participant selected a war congruent statement together with its war incongruent counterpart. Inconsistent responses/trials were then excluded from statistical analysis because these could not provide a reliable indication for people’s endorsement of war congruent inclinations/scenarios.

The subsequent coding steps were therefore applied only to consistent choices/trials. Since for each trial, participants had to select 3 options, and since war congruent options were coded as 1, each consistent trial could receive a score that ranged from 0 (for selection of 3 war incongruent options) to 3 (for selection of 3 war congruent options). Afterwards, for each participant, a total score was computed by adding the scores of each trial. Since each participant saw 6 passages/trials, they were assigned a total score that could range from 0 to 18.

As for the set of socio-demographic measures, participants’ education level was coded into 4 categories, based on the Italian education system: “A” for *Licenza Media* (i.e., middle school diploma); “B” for *Diploma di Scuola Media Superiore* (i.e., secondary school certificate); “C” for University student (i.e., students attending a University programme and not yet graduated); and “D” for University Degree (i.e., individuals who obtained a graduation).

Italy is divided into 20 Regions and, to handle the data, the Regions were classified into 3 categories, based on the level of risk of the SARS-CoV-2 spread at the time the questionnaire has been distributed (June 2020): Lombardy, Emilia-Romagna and Piedmont were coded as High-Level risk (i.e., High); Veneto, Liguria and Tuscany as Medium-Level risk (i.e., Medium); all other Regions as Low-Level risk (i.e., Low; in our sample: Calabria, Sicily, Sardinia, Campania, Puglia and Friuli Venezia Giulia).

Two composite scores for participants’ sources of information were computed: *TV_Newspaper* was obtained by adding participants’ frequency rating (i.e., from 1- never to 5-always) for the traditional sources of information (news/programs on television and newspapers); *Ind_Social* was obtained by adding participants’ frequency rating for independent information channels and social networks.

### Statistical analyses

Data were analyzed as follows. First, to investigate any potential effect of the war metaphor framing, a between-condition analysis was conducted on participants’ total score in the metaphor task using inferential non-parametric statistics (i.e., Wilcoxon signed-rank test) that compared the total score between participants who saw the neutral condition and those who saw the war metaphoric condition.

Second, for each of the socio-demographic measures, between-group analyses were conducted to estimate any group differences comparing the test score/measure between participants in the neutral condition and those in the metaphoric condition. A Wilcoxon signed rank test statistics was conducted on the following between-group differences: age, TV_Newspaper, Ind_Social, and each of the 5 measures for political inclination (i.e., political orientation, Premier’s approval, national government’s approval, opposition’s approval, and region governor’s approval). A chi-square statistics with Yate’s continuity correction was run to estimate between-group differences for gender, education level and Region SARS-CoV-2 risk.

Finally, multiple regression analyses were carried out to investigate the impact of the collected socio-demographic measures on participants’ sensitivity to the war metaphor framing with respect to the pandemic situation. The regression models included participants’ scores in the metaphor task as the outcome variable and the socio-demographic measures as predictors, as well as any resulting interactions. Given the exploratory nature of these analyses and the absence of previous experimental literature on the same topic, a backward method based on AIC values was applied to determine the influence of each predictor within the regression model(s). Multicollinearity (VIF and mean VIF) and auto-correlation (Durbin-Watson test) diagnostics were carried out for each regression model. In addition, casewise statistics was conducted to treat potential influential cases in the models. Potential large residuals were investigated by fixing the standardized residuals within ±2 and looking at their Cook’s distance, average leverage values and upper and lower limit of acceptable values for the covariance ratio.

All statistical analyses were conducted using R software [40]. Data are available on the Open Science Framework web platform, comprising the R code used for the analysis as well as the details of the casewise statistics (Link to the project: https://osf.io/2yrxa/?view_only=df07e8cc3a9e4ca097c55e7ddd7f380c).

## Results

### Socio-demographic assessment

Table 1 reports the descriptive statistics (i.e., frequency or mean(SD), depending on the measure) for each of the socio-demographic measures collected, both in total and by experimental condition metaphoric vs. neutral. Inferential statistics on each of the socio-demographic measures collected revealed no significant differences between the group of participants who were administered the passages in the neutral condition and the group of participants who were administered the passages in the war metaphoric condition (all p_s_ = n.s.), thus suggesting that the two groups did not differ in terms of socio-demographic characterization. All statistical details are reported in Table 1, too.

**Table 1:** Descriptive and inferential statistics for the set of socio-demographic measures.

**Socio-demographic measure**		**Frequency (%) (N. of counts)**			**Test Statistics**	
		**ToT**	**Condition Metaphor**	**Condition Neutral**	**Chi-square**	**p**
Gender	*F*	64.5 (129)	63.7 (70)	61.5 (59)	χ ^2^(1) = 0.74	.38
	*M*	35.5 (71)	32.7 (34)	38.5 (37)		
Education Level	*A*	4.5 (9)	2.8 (3)	6.3 (6)	χ ^2^(3) = 6.05	.10
	*B*	20.8 (42)	23.6 (25)	17.7 (17)		
	*C*	57.4 (116)	61.3 (65)	53.1 (51)		
	*D*	17.3 (35)	12.3 (13)	22.9 (22)		
Region SARS-CoV-2 Risk	*High*	84.9 (169)	86.5 (90)	83.2 (79)	χ ^2^(2) = 0.87	.64
	*Medium*	9.5 (19)	7.7 (8)	11.6 (11)		
	*Low*	5.5 (11)	5.8 (6)	5.3 (5)		
**Socio-demographic measure**		**Mean (SD)**			**Test Statistics**	
		**ToT**	**Condition Metaphor**	**Condition Neutral**	**Wilcoxon (W)**	**p**
Age		33.53(14.77)	32.54(14.63)	34.62(14.93)	4733.5	.39
TV_Newspaper		6.41(1.80)	6.41(1.94)	6.41(1.65)	3839.5	.85
Ind_Social		6.04(2.03)	6.13(2.02)	5.94(2.05)	3932	.64
Political orientation		3.04(1.77)	3.02(1.88)	3.05(1.66)	3632	.64
Approval Premier		6.94(1.84)	6.95(1.66)	6.94(2.03)	3671.5	.73
Approval Government		5.92(1.90)	5.86(1.90)	5.98(1.91)	3530	.44
Approval Opposition		3.54(1.96)	3.55(2.04)	3.53(1.89)	3770.5	.97
Approval Region Governor		5.81(2.88)	6.01(2.90)	5.95(2.87)	4137.5	.27

Gender, Education level (A: middle school diploma; B: secondary school certificate; C: University student; D: Degree) and Region SARS-CoV-2 Risk (High, Medium, Low): frequency distribution in total and by condition; chi-squared statistics for differences between conditions. All other measures: mean (SD) in total and by condition; Wilcoxon signed rank test statistics for differences between conditions.

### Metaphor results and analyses of predictors

The total score for the metaphor task was on average 3.37(2.16) for the metaphor condition and 3.04(2.28) for the neutral condition. Statistical analyses revealed no significant differences between conditions (W = 5557.5; p = .25). Therefore, people’s sensitivity to war congruent claims about the SARS-CoV-2 pandemic does not seem to be influenced by the war metaphoric framing *per se*.

**Fig 2:**
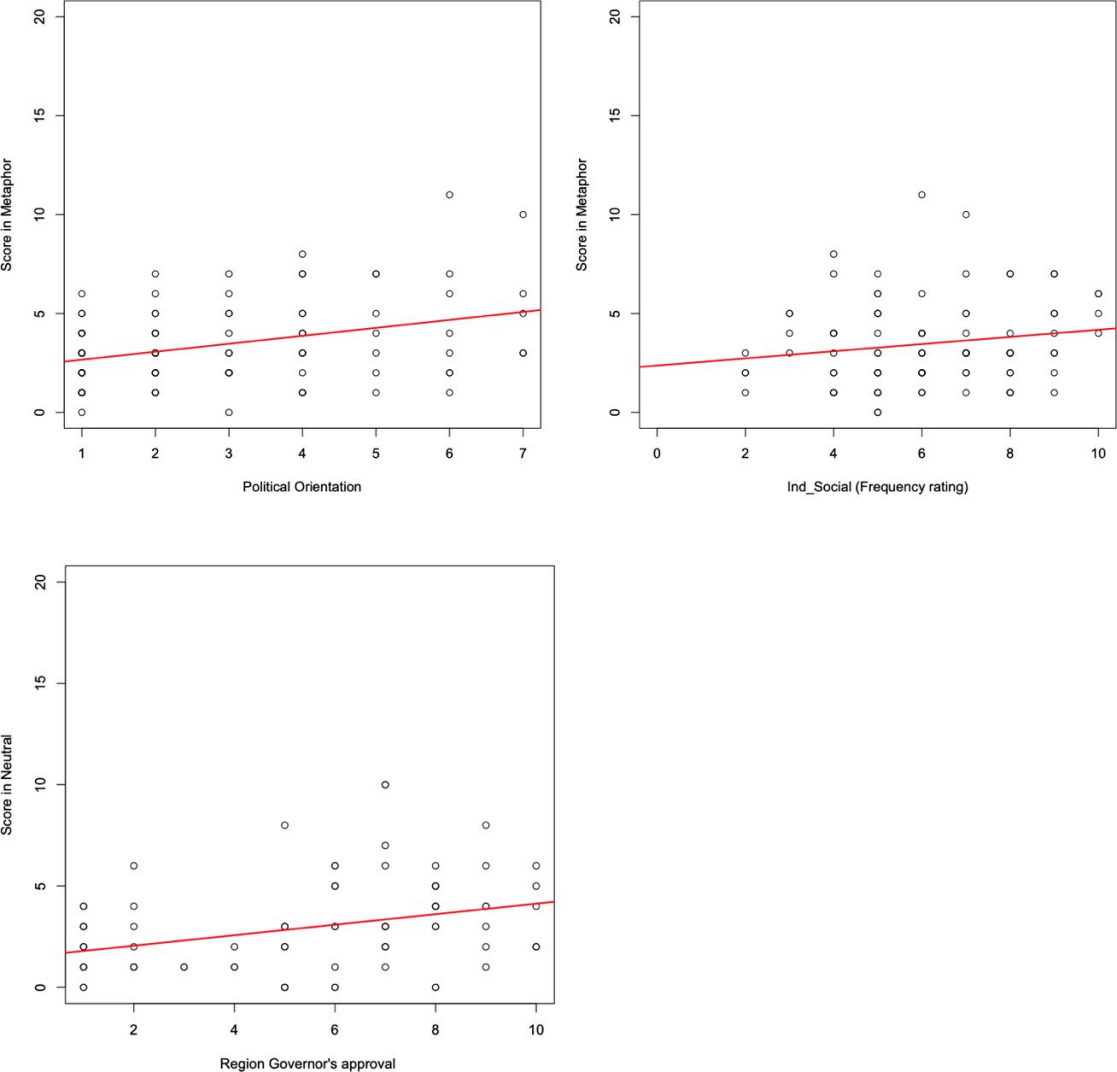
Predictors of participants’ score in the metaphor task. Top: Correlations between participants’ score in the metaphor condition and their political orientation (left panel) and the frequency rating (composite score) for independent information channels and social networks (right panel). Bottom: Correlation between participants’ score in the neutral condition and their Region governor’s approval.

Most important for the purpose of this study, interesting patterns of results emerged from the analyses of predictors. The resulting statistics of the final backward multiple regression models are reported in Tables 2–4, together with details on the diagnostic tests for multicollinearity and auto-correlation. A first multiple regression analysis was conducted on the total score in the metaphor task from the two groups of participants (i.e., condition neutral vs. metaphoric), with a model that included as predictors condition, the socio-demographic measures and their interaction. This first model was affected by autocorrelation and multicollinearity. For completeness of information, the statistical details of this model are reported in Table 2. As shown in Table 2, condition is the most likely responsible for the model multicollinearity (see mean VIF of the model and GVIFs values for condition in Table 2). Therefore, to deal with multicollinearity and obtain reliable results, we conducted separate regression models by condition. For these models, we decided not to include the Region SARS-CoV-2 Risk as a predictor because this was an approximate classification drawn by the authors and therefore did not provide a precisely quantified measure for the regional spread of the pandemic. Thus, before undergoing the backward procedure, each of the two models included: participants’ total score in, respectively, the metaphoric and neutral condition as the outcome variable, and the following socio-demographic measures as the predictors: Gender, Age, Education level, TV_Newspaper, Ind_Social, Political orientation, approval of Premier, national government, opposition and Region Governor. Interestingly, these models revealed two different patterns of results in the two groups of participants. In fact, the regression statistics on participants’ total score in the metaphoric condition revealed that this was significantly predicted by political orientation and the degree to which people relied on independent information channels and social networks as their source of information (Table 3). Specifically, political orientation impacted the most our participants’ selection of war congruent options when reading a metaphoric passage on the pandemic (β = .38; p < .001): the more on the right the political orientation, the more war congruent options were selected. This was followed by the source of information *Ind_Social* (β = .20; p = .03), for which a positive correlation emerged, thus indicating that the more frequently independent information channels and social networks were used as source of information, the more war congruent options were selected in the metaphoric condition–Fig 2 (top panel).

**Table 2:** Multiple regression statistics for the final overall model.

Overall Model		Start AIC	Final AIC	F	R^2^	ΔR^2^	p			
		282.63	268.46	(20,162) = 3.26	0.28	0.19	< .0001*			
*Coefficients*		Estimate (*B*)	Std. Error (*B*)	t	p	β	(G)VIF	GVIF^1/(2*df)^	Mean (G)VIF	Auto-correlation
Intercept		0.66	2.19	0.30	.76	0				
Condition		-1.06	3.25	-0.32	.74	-0.24	124.54	11.15		
Gender		0.49	0.32	1.53	.12	0.10	1.12	1.06		
Age		-0.02	0.02	-1	.31	-0.19	8.42	2.90		
Education level	B	-0.98	1.30	-0.75	.44	-0.17	47.38	1.90		
	C	-1.90	1.59	-1.20	.23	-0.42				
	D	-0.73	1.34	-0.54	.58	-0.12				
Region Covid Risk	Low	-0.66	0.64	-1.02	.30	-0.07	1.29	1.06		
	Medium	-1.61	0.53	-3.01	.002*	-0.21				
TV_Newspaper		0.11	0.11	1.02	.30	0.09	1.92	1.38		
Ind_Social		0.15	0.07	2.03	.04*	0.14	1.14	1.06		
Political Orientation		0.46	0.12	3.77	.0002**	0.38	2.35	1.53		
Approval Premier		0.20	0.08	2.39	.01*	0.17	1.28	1.13		
Approval Opposition		-0.13	0.08	-1.58	.11	-0.12	1.44	1.20		
Approval Region Governor		0.19	0.06	3.27	.001***	0.26	1.46	1.20		
Condition:Age		0.06	0.04	1.35	.17	0.59	43.36	6.58		
Cond:Education	Cond:B	2.78	1.84	1.51	.13	0.35	1030.73	3.17		
	Cond:C	3.50	2.42	1.44	.14	0.70				
	Cond:D	0.94	1.91	0.49	.62	0.13				
Condition:TV_Newspaper		-0.45	0.17	-2.57	.01*	-0.70	17.05	4.12		
Condition:Political Orientation		-0.42	0.16	-2.56	.01*	-0.38	5.20	2.28		
									30.01	DW = 1.71; p = .04

The table displays the multiple regression statistics for the final overall model (i.e., outcome variable: Total score in conditions metaphoric and neutral of the metaphor task) as resulting from the backward procedure and coefficient values.

**Table 3:** Multiple regression statistics of the metaphoric model.

Model Metaphoric	Start AIC	Final AIC	F	R^2^	ΔR^2^	p		
	135.43	125.72	(4,89) = 7.27	0.24	0.21	< .0001***		
*Coefficients*	Estimate (*B*)	Std. Error (*B*)	t	p	β	VIF	Mean VIF	Autocorrelation
Intercept	-1.59	1.16	-1.37	.17	0			
Ind_Social	0.21	0.09	2.18	.03*	0.20	1.04		
Political orientation	0.44	0.11	3.84	.0002***	0.38	1.17		
Approval Premier	0.23	0.12	1.88	.06	0.18	1.10		
Approval Region Governor	0.12	0.07	1.78	.07	0.17	1.08		
							1.10	DW = 1.85; p = .46

The table reports the multiple regression statistics separated by condition as resulting from the backward procedure and coefficient values. Model Metaphoric: outcome variable = total score in condition metaphor.

**Table 4:** Multiple regression statistics of the neutral model.

Model Neutral	Start AIC	Final AIC	F	R^2^	ΔR^2^	p		
	149.59	137.75	(2,86) = 5.29	0.12	0.10	.002**		
*Coefficients*	Estimate (*B)*	Std. Error (*B*)	t	p	β	VIF	Mean VIF	Autocorrelation
Intercept	3.16	0.98	3.21	.001***	0			
TV_Newspaper	-0.25	0.13	-1.82	.07	-0.18	1		
Approval Region Governor	0.25	0.07	3.22	.001***	0.32	1		
							1	DW = 1.74; p = .23

The table reports the multiple regression statistics separated by condition as resulting from the backward procedure and coefficient values. Model Neutral: Outcome variable = total score in condition neutral.

Conversely, a different picture emerged in the predictors analysis on the total score for the neutral (i.e., non-metaphorical) passages (Table 4). Here, only the region governor’s approval significantly predicted people’s score in the neutral condition of the metaphor task (β = .32; p = .001): the higher the participants’ approval of their region governor, the higher their score, i.e., more war congruent options after reading a non-metaphoric passage on the pandemic–Fig 2 (bottom panel).

## Discussion

The aim of this study was to assess whether the presentation of the COVID-19 pandemic within a war metaphor increased participants’ willingness to prefer war congruent options compared to the presentation of the same scenario in neutral terms. Since there was not a statistically significant difference in participants’ choices between conditions, we did not find evidence of a metaphorical framing effect *per se*. Quite interestingly, though, we did find an effect on specific categories of individuals: participants who declared to be right-winged and those who gather information relying on independent information channels and on social networks opted more for war congruent options after reading the passages with the war metaphorical framing.

As for the effect of political orientation, our results at least apparently contrast with Thibodeau and Boroditsky’s [31, 32] (see also [36]). They found that Republicans were less likely than Democrats and Independents to be influenced by the “crime is a beast” metaphor: even if in general Republicans were preferring more enforcement-oriented measures, Democrats and Independents tended to provide more metaphor congruent options. To account for this asymmetry, the authors suggest that individuals are more resistant to persuasion when they are already ideologically committed to an issue–as Republicans might be on crime reduction programs, or Democrats on carbon reduction programs, see [41]. This explanation does not seem viable for our study, since the best way to handle a pandemic does not appear to be ideologically grounded.

Concerning the role of the sources of information on the choice of metaphor congruent responses, we believe two different lines of explanations can be proposed. On the one hand, within the Conceptual Metaphor Theory, what needs to be explained is (not that some participants are influenced by the war metaphor, but) that other individuals, those who gather information mainly on traditional channels (TV and newspapers), do not seem to be prominently influenced by the war metaphor framing effect. Assuming that traditional channels are those that transmit complete and reliable information, one can conclude that those participants were already competent enough on SARS-CoV-2 issues, and therefore less prone to be influenced by the metaphorical framing effect, as argued by Robins & Mayer [42]. They proposed the metaphor termination hypothesis: those who are competent on a given issue will be less influenced by a metaphor. Since metaphor is an effective tool to understand a hitherto unknown concept or issue, by making it more concrete and comprehensible, it becomes “unnecessary in cases in which […] a reasoner can use existing domain general knowledge to understand the situation” [42: 61]; thus, participants who are already competent on that issue are less prone to be influenced by the metaphorical framing effect compared to those with reduced knowledge (see also [43]). Importantly, this approach assumes the existence of the metaphorical framing effect, that can be attenuated for specific categories of individuals.

We propose, instead, to walk a different path, namely one that shifts the perspective from asking who might be immune to the frame effect to the question of who might be influenced by a metaphor. In other words, we defend an approach more *à la* Sontag, in which metaphors are rhetoric tools that might induce persuasive effects on some groups of people, and we argue against a strictly psychological cognitive account that views metaphors as shaping our thoughts and actions, and against which only some categories of persons might be immunized. Let us start by reconstructing the argument behind the cognitive approach to metaphors, and let us break up the metaphorical framing effect into the several steps required to influence the interlocutors’ behavior. (i) Once the speaker utters a metaphor to talk about an issue, if the metaphor is structural, then also the other entailments connected to the frame are activated and become salient. (ii) These additional entailments (might) affect the interlocutors’ beliefs and their opinion formation about the framed issue. (iii) The newly created beliefs and opinions (might) enter into people’ inferences about the framed problem. (iv) Finally, these additional evoked entailments (might) affect interlocutors’ decisional processes, hence, their practical attitudes and actions. We believe that there is a sort of ambiguity in the way this argument is presented.

Even if in academic papers the transition from (i) to (iv) is correctly depicted as a series of *possible* steps (that is, with the presence of the modal *might*), we believe that asking what individual factors might block the framing effect presupposes the inevitability of the transition from (i) to (iv) (that is, without the *might*). This line of reasoning would represent a slippery slope argument, that starts from the plausible premises (i) and (ii), but then becomes fallacious when it assumes a faulty generalization about the predicted perlocutionary effects that a speech act involving a (conceptual) metaphor may have upon the listeners. We contend that asking which factors might prevent interlocutors from the metaphorical framing effect is tantamount as assuming that they will be inevitably conditioned by a metaphor (and thus somehow forced to come to believe the related entailments, and eventually act accordingly) unless some factors (e.g., knowledge about the framed issue) protect them. On the contrary, we believe that the question to pose is what factors might persuade a person to accept the additional entailments evoked by a structural frame, and possibly to act accordingly.

In particular, in our experiment, only those participants who declared to endorse right-wing political positions, and only those who gather their information from independent information channels and social media were indeed influenced by the war metaphor, and opted for more war congruent options in the metaphorical condition. This suggests that the activation of bellicose metaphors is effective only on those individuals, and not in general.

Studies on persuasion and social judgments extensively argued that in general people tend to accept new information when it is consistent with other things they believe to be true (see [44] and [45] for a review). The metaphorical framing effect was found to be amplified for those participants who had personal interest in the domain: individuals who enjoy sports were more sensitive to the argument strength of arguments with sports metaphor [46], and citizens with aggressive personality traits expressed significantly greater support for political violence when they were exposed to political messages infused with violent metaphors [47]. The bellicose-related options that were presented in the questionnaire depicted outcomes that are generally accepted in the right-wing culture, such as authoritarianism (see [48]), the willingness to suspend civil liberties for greater personal safety and security, and even an aggressive reaction against minorities who might challenge the Nation security [49, 50]. Therefore, we hypothesize that participants with a right-wing political orientation tended to share a set of beliefs that were consistent with the war metaphor congruent options. Hence, when presented with the bellicose metaphor, also other evoked entailments were easily added to these participants’ set of beliefs, entering their inferential reasoning and eventually increasing the likelihood of military-like statements.

As for the increase in the choice of war metaphor congruent options for those participants who gather information through independent channels and social networks, we hypothesize that the reason for their vulnerability to the metaphorical framing effect is again couched in general mechanisms that facilitate the acceptance of new information. However, we propose that here the mechanisms are not based on consistency with previous beliefs, but with concordance with arousal levels. A growing body of research established that there is a link between the virality, hence the diffusion, of the messages spread through unconventional channels (blogs, YouTube, social networks) and their capacity to stimulate emotions [51]. In particular, news is more easily shared when it transmits negative emotions (such as anger and disgust) [52, 53]. Evoking war scenarios to talk about the pandemic increases the level of emotional involvement. This strategy becomes more effective on those participants who are more interested in high level of arousals, such as those who use independent information channels and social networks, and, in our view, these individuals will be more susceptible to accept also the other bellicose entailments that the war metaphor evokes.

To sum up, we believe that structural metaphors may indeed evoke a set of related entailments, but we propose that the propositions made salient through a structural metaphor will be believed only by those individuals whose prior set of personal opinions is already consistent with these (such as right-winged individuals), or by those who are attracted by statements that transmit high level of arousals (such as those who use unconventional media) (see also [35]). Therefore, structural metaphors might have a cognitive import only when they accomplish a rhetorical function: a structural metaphor will influence an individual’s reasoning only when the metaphorical cluster coheres and provides a frame that is congruent with that person’s inclinations and opinions.

Finally, let us mention one more result of our study: when presented with the neutral version of the stories (those that did not contain any metaphorical expression), participants who expressed their approval towards their Region governor (who is in charge of the local sanitary system) tended to opt more for the “negative” options related to the war metaphorical scenario. This is not at all related to the Conceptual Metaphor Theory–since no metaphors were involved. The interpretation of this result is quite straightforward: the options that were presented as congruent with the war scenarios involved, among other things, the acceptance of authoritarian decisions and of the (temporarily) suspension of civil liberties for security reasons. It is important to highlight that individuals may endorse these options for independent reasons that are not connected to the war metaphor frame, such as their personal approval of the governor’s capacity to handle the pandemic. In Thibodeau and Borodistky studies, the options related to enforcement measures (viewed as congruent with the “crime is a beast” scenarios) were in fact chosen by most of the participants who were exposed to the “crime is a virus” version of the story, and the metaphorical effect consisted in the relative increase or decrease of the metaphorical congruent alternatives in the two versions. Overall, these patterns of results highlight the importance of an individual’s set of opinions and preferences in the choice of their preferred actions, and the possible influence of metaphorical frames has to be weighted against this preexistent set of beliefs.

## Conclusions

In our experiment, we adopted the line of reasoning of the many journalists and academics who feared that the use of a war metaphor to talk about the SARS-CoV-2 contagion might have negative outcomes in the audience, leading citizens to endorse coercive opinions and to prefer conflictual practical solutions to solve the SARS-CoV-2 problem.

Our results suggest that this is not the case: when the COVID-19 issues were talked about with military metaphors, participants were not overall more inclined to prefer bellicose options. Still, we did find that the metaphorical framing effect appeared to influence some individuals’ choices. In light of this, we are sympathetic with some intuitions of the Conceptual Metaphor Theory. In particular, we agree that structural metaphors may contribute to trigger sets of salient conceptual entailments via the activation of the relevant frame. Also, we recognize that, besides their cognitive import, conceptual metaphors serve an emotive function, namely, they can affect language users’ emotive states. Conversely, we are more skeptical about the hierarchically superior explanatory role that Conceptual Metaphor Theory attributes to metaphors in explaining language users’ opinion formation and consequent behavior. We showed that the metaphor framing effect is not manifest in all individuals, rather it is mediated by, and subordinated to, psycho-social variables such as participants’ political preferences and reading habits. We hypothesized that the newly activated metaphor-related entailments are effective only for those individuals whose cognitive and emotional states are coherent with these.

Still, this study cannot establish whether the metaphor framing effect, that ought to persuade interlocutors to act according to the entailments made salient by the activation of the structural metaphor, was blocked also for other reasons. In fact, our results cannot exclude the possibility that, the newly created beliefs were added indeed to the individuals’ set of beliefs, but did not influence the speakers’ decisional processes and behavioral attitudes. Even if language users were to endorse a new opinion through a metaphorical framing effect, this does not ensure that they are disposed to act accordingly. The switch from entertaining a (coercive, conflicting) belief to adopt (coercive, conflicting) behaviors may be affected by forms of cognitive dissonance; e.g., if a language user holds conflicting or contradictory beliefs or opinions, then it is difficult to determine which one takes the priority and actually guides their decisional processes. To conclude, future research should investigate in depth the various steps that are required for a structural metaphor to influence the interlocutors’ formation of new beliefs, and their willingness to act accordingly, by taking into serious consideration the mediation of individual psycho-social characteristics.
